# Supplementary material for: Segmenting accelerometer data from daily life with unsupervised machine learning
Source: PLoS One. 2019 Jan 9;14(1):e0208692. doi: 10.1371/journal.pone.0208692 (PMC6326431; doi:10.1371/journal.pone.0208692)
Supplement: S1 Appendix — (PDF) [file pone.0208692.s001.pdf]

S1 Appendix: supplementary results

The states of the *acceleration+angles* models are described in detail here:

- State A and B are the least active state with acceleration values close to zero (<1m*g*) and the longest durations. The difference is the *angle-z* value, that has mostly positive values for state B and negative values for state A. The *angle-x* values in both states have a relative large spread. Further, a strong agreement is observed between time spent in cut-points category <1- sustained activity> and the time spent in states A and B, see Table 2.
- State C, D, E and F have low acceleration levels (means around 6-10 m*g*), durations that are shorter than the inactive A and B states and are mostly present in the inactive cut-points categories (2, 3 and 4). The difference between states C, D, E and F is mostly in the angles:
    - State D has higher angle-x values and a large spread in angle-y values
    - State E and F have very low spread in the angle-y values, with mostly values around zero.
    - State E has mostly positive angle-z values, state D and F mostly negative angle-z values and state C has angle-z values around zero.
- State G, H and I have comparable acceleration levels with a mean around 50m*g*, which seems to fall in between the ranges considered for inactivity and light activity (LPA categories). State H has the shortest durations. State G has higher angle-x values, a larger spread in angle-y values and more positive angle-z values. State I has mostly negative angle-y values a smaller spread in angle-z.
- State J is the most active state, having a mean acceleration of around 300m*g*. This state is mostly present in the cut-points derived MVPA categories and has a short duration.
