# Supplementary material for: Segmenting accelerometer data from daily life with unsupervised machine learning
Source: PLoS One. 2019 Jan 9;14(1):e0208692. doi: 10.1371/journal.pone.0208692 (PMC6326431; doi:10.1371/journal.pone.0208692)
Supplement: S1 Table — (PDF) [file pone.0208692.s002.pdf]

S1 Table

Average time spent (minutes) per participant per day in each state from the acceleration method (sorted by mean acceleration) and cut-points category

cut-points category	state											
	**A**	**B**	**C**	**D**	**E**	**F**	**G**	**H**	**I**	**J**	**total**	**Avg acc**
**1 - sustained activity**	430	0	0	0	0	2	0	0	0	0	432	0.0
**2 - non-bouted inactivity**	3	82	62	96	8	14	2	16	2	1	284	14.5
**3 - 10-30min inactivity**	3	88	40	16	0	8	0	1	0	0	156	13.3
**4 - >=30min inactivity**	5	164	65	29	0	15	0	1	0	1	280	13.3
**5 - non-bouted LPA**	0	0	8	51	6	23	5	39	8	2	142	71.6
**6 – 1-10min LPA**	0	0	0	4	3	1	3	5	0	0	15	75.1
**7 - >=10min LPA**	0	0	0	0	0	0	0	0	0	0	0	77.3
**8 - non-bouted MVPA**	0	0	0	2	0	12	2	23	8	9	57	220.4
**9 - 1-10min MVPA**	0	0	0	0	0	2	0	5	13	7	28	286.7
**10 - >=10min MVPA**	0	0	0	0	0	2	0	2	9	5	17	310.5
**total**	441	334	174	198	17	79	12	92	40	24	1412	
**avg acc**	0.0	5.2	19.0	35.4	46.9	78.4	87.3	100.1	172.4	483.3		
