# Supplementary material for: Segmenting accelerometer data from daily life with unsupervised machine learning
Source: PLoS One. 2019 Jan 9;14(1):e0208692. doi: 10.1371/journal.pone.0208692 (PMC6326431; doi:10.1371/journal.pone.0208692)
Supplement: S2 Table — (PDF) [file pone.0208692.s003.pdf]

S2 Table

Average time spent (minutes) per participant per day in each state (acceleration method) and the top 10 activities, acceleration model

	state										
	**A**	**B**	**C**	**D**	**E**	**F**	**G**	**H**	**I**	**J**	**total**
**Sleeping and resting (including sick in bed)**	274.2	91.7	14.4	20.2	1.0	14.0	1.2	8.9	3.0	1.4	429.9
**In class**	3.4	18.9	25.1	24.7	1.0	7.0	0.7	11.9	5.2	2.2	99.9
**Watch TV, DVDs, downloaded videos**	11.8	31.4	15.2	15.0	0.8	6.1	0.7	5.3	1.9	1.3	89.4
**Speaking, socialising face-to-face**	4.1	14.5	10.2	12.3	1.1	4.3	0.8	5.4	2.4	1.4	56.4
**Personal care (including taking a shower/bath, grooming, getting dressed etc.)**	7.9	11.7	6.9	9.5	1.0	3.4	0.6	5.5	2.0	1.0	49.4
**Eating a meal**	3.0	13.4	9.2	10.4	0.8	3.7	0.6	4.3	2.0	1.2	48.6
**Playing electronic games and Apps**	4.9	15.4	11.3	8.3	0.4	3.4	0.1	2.2	0.7	0.9	47.6
**Other activities not listed**	3.3	6.3	5.0	7.6	0.9	2.5	0.5	3.6	1.7	0.9	32.2
**Homework**	2.7	8.8	6.8	5.6	0.5	2.1	0.2	1.6	0.6	0.6	29.6
**Travel by car, van (including vehicles owned by friends and family)**	1.5	4.9	6.0	6.1	2.2	2.0	0.5	3.0	1.4	0.9	28.6
