# Supplementary material for: Segmenting accelerometer data from daily life with unsupervised machine learning
Source: PLoS One. 2019 Jan 9;14(1):e0208692. doi: 10.1371/journal.pone.0208692 (PMC6326431; doi:10.1371/journal.pone.0208692)
Supplement: S3 Table — (PDF) [file pone.0208692.s004.pdf]

S3 Table

Kullback-Leibler divergences for the distributions on the full model and a subset model

	full 500			sub 250			Kullback-Leibler divergence	
**state**	**acc mean**	**acc sigma**	**dur lmbda**	**acc mean**	**acc sigma**	**dur lmbda**	**acceleration**	**duration**
**A**	0.0166	0.0002	118.7	0.0400	0.0005	111.4	0.01	0.34
**B**	5.22	0.02	28.7	5.81	0.02	27.8	0.00	0.02
**C**	18.96	0.25	61.8	20.98	0.25	104.0	0.28	14.48
**D**	35.37	0.91	23.4	29.14	0.59	20.7	0.09	0.23
**E**	46.9	0.6	144.2	63.2	1.5	141.9	0.01	0.03
**F**	78.4	4.1	2.8	71.4	3.3	2.2	7.53	0.11
**G**	87.3	2.1	165.3	88.8	2.5	91.6	0.78	34.43
**H**	100.1	4.1	53.3	104.4	4.5	35.7	0.32	5.47
**I**	172.4	9.8	57.5	199.5	13.5	34.5	0.99	9.15
**J**	483.3	226.8	3.9	547.7	273.4	3.1	312.68	0.14
