# Supplementary material for: Segmenting accelerometer data from daily life with unsupervised machine learning
Source: PLoS One. 2019 Jan 9;14(1):e0208692. doi: 10.1371/journal.pone.0208692 (PMC6326431; doi:10.1371/journal.pone.0208692)
Supplement: S4 Table — (PDF) [file pone.0208692.s005.pdf]

S4 Table

Distribution parameters of the HSMM (acceleration) model, for each state

state	acceleration		duration
	mean	sigma	lambda
A	0.000	2.50E-07	594 sec
B	0.005	1.70E-05	144 sec
C	0.019	2.50E-04	309 sec
D	0.035	9.10E-04	117 sec
E	0.047	6.40E-04	721 sec
F	0.078	4.10E-03	14 sec
G	0.087	2.10E-03	827 sec
H	0.100	4.10E-03	267 sec
I	0.172	9.80E-03	287 sec
J	0.483	2.30E-01	19 sec
