# Supplementary material for: Segmenting accelerometer data from daily life with unsupervised machine learning
Source: PLoS One. 2019 Jan 9;14(1):e0208692. doi: 10.1371/journal.pone.0208692 (PMC6326431; doi:10.1371/journal.pone.0208692)
Supplement: S5 Table — (PDF) [file pone.0208692.s006.pdf]

S5 Table

Distribution parameters of the HSMM (acceleration+angles) model for each state

	**acceleration**		**anglex**		**angley**		**anglez**		**duration**
**state**	**mean**	**sigma**	**mean**	**sigma**	**mean**	**sigma**	**mean**	**sigma**	**lambda**
**A**	0.000	5.5E-07	5	0.0002	7	0.0001	-48	-0.0001	584 sec
**B**	0.001	2.7E-06	29	0.0006	-11	-0.0059	39	-0.0045	393 sec
**C**	0.007	6.3E-05	-35	-0.0097	45	-0.0083	8	-0.0027	77 sec
**D**	0.008	5.8E-05	19	0.0025	16	-0.0191	-37	-0.0023	86 sec
**E**	0.008	1.3E-04	-51	-0.0058	-2	0.0046	35	-0.0103	69 sec
**F**	0.010	1.3E-04	-51	0.0147	4	-0.0018	-34	-0.013	75 sec
**G**	0.045	2.4E-03	21	-0.1807	-2	-0.0191	33	-0.2734	60 sec
**H**	0.056	1.7E-03	-37	-0.0685	13	0.0055	-13	0.0509	16 sec
**I**	0.060	4.4E-03	-39	0.0099	-40	0.0355	-3	0.1478	87 sec
**J**	0.309	1.2E-01	-28	-0.0301	-3	-1.83	2	0.2618	20 sec
